# Automated detection of cerebral microbleeds on T2*-weighted MRI

**DOI:** 10.1038/s41598-021-83607-0

**Published:** 2021-02-17

**Authors:** Anthony G. Chesebro, Erica Amarante, Patrick J. Lao, Irene B. Meier, Richard Mayeux, Adam M. Brickman

**Affiliations:** 1 Taub Institute for Research on Alzheimer’s Disease and the Aging Brain, Vagelos College of Physicians and Surgeons, Columbia University, New York, NY USA; 2 Gertrude H. Sergievsky Center, Vagelos College of Physicians and Surgeons, Columbia University, New York, NY USA; 3 Department of Neurology, Vagelos College of Physicians and Surgeons, Columbia University, 630 West 168th Street, PS Box 16, New York, NY 10032 USA

**Keywords:** Software, Cerebrovascular disorders, Diagnostic markers

## Abstract

Cerebral microbleeds, observed as small, spherical hypointense regions on gradient echo (GRE) or susceptibility weighted (SWI) magnetic resonance imaging (MRI) sequences, reflect small hemorrhagic infarcts, and are associated with conditions such as vascular dementia, small vessel disease, cerebral amyloid angiopathy, and Alzheimer’s disease. The current gold standard for detecting and rating cerebral microbleeds in a research context is visual inspection by trained raters, a process that is both time consuming and subject to poor reliability. We present here a novel method to automate microbleed detection on GRE and SWI images. We demonstrate in a community-based cohort of older adults that the method is highly sensitive (greater than 92% of all microbleeds accurately detected) across both modalities, with reasonable precision (fewer than 20 and 10 false positives per scan on GRE and SWI, respectively). We also demonstrate that the algorithm can be used to identify microbleeds over longitudinal scans with a higher level of sensitivity than visual ratings (50% of longitudinal microbleeds correctly labeled by the algorithm, while manual ratings was 30% or lower). Further, the algorithm identifies the anatomical localization of microbleeds based on brain atlases, and greatly reduces time spent completing visual ratings (43% reduction in visual rating time). Our automatic microbleed detection instrument is ideal for implementation in large-scale studies that include cross-sectional and longitudinal scanning, as well as being capable of performing well across multiple commonly used MRI modalities.

## Introduction

Microhemorrhages in the brain, known as cerebral microbleeds, are small, persistent deposits of products from blood breakdown, primarily hemosiderin, which have been contained in perivascular regions by macrophages^1–9^. Radiologically, microbleeds are identified on T2*-weighted magnetic resonance imaging (MRI) scans as roughly spherical signal voids, or hypointensities, due to the strong paramagnetic properties of the hemosiderin left after a bleed has occurred^2–4,6–8,10,11^. Cerebral microbleeds are associated with a number of outcomes, such as small vessel disease^3,10,12^, stroke^8,9,13^, traumatic brain injury^14^, radiation-induced bleeding^15–17^, cognitive decline^2,11,18^ and vascular dementia^10,11^. Lobar distributions of cerebral microbleeds are considered markers of cerebral amyloid angiopathy^1^, and are a prominent feature of Alzheimer’s disease (AD)^4,19,20^. In addition to signaling vascular forms of amyloid pathology, particularly in AD, microbleeds have emerged as a pernicious side effect of anti-amyloid treatments, so-called amyloid related imaging abnormalities related to hemosiderin deposits (ARIA-H)^4^, a necessary and important consideration in the enrollment of participants into AD therapeutic trials^4,21,22^. Although microbleeds can be present asymptomatically, early detection can be crucial in estimating risk for later cerebrovascular disease and cognitive decline^2,3,11,18^.

**Figure 1:**
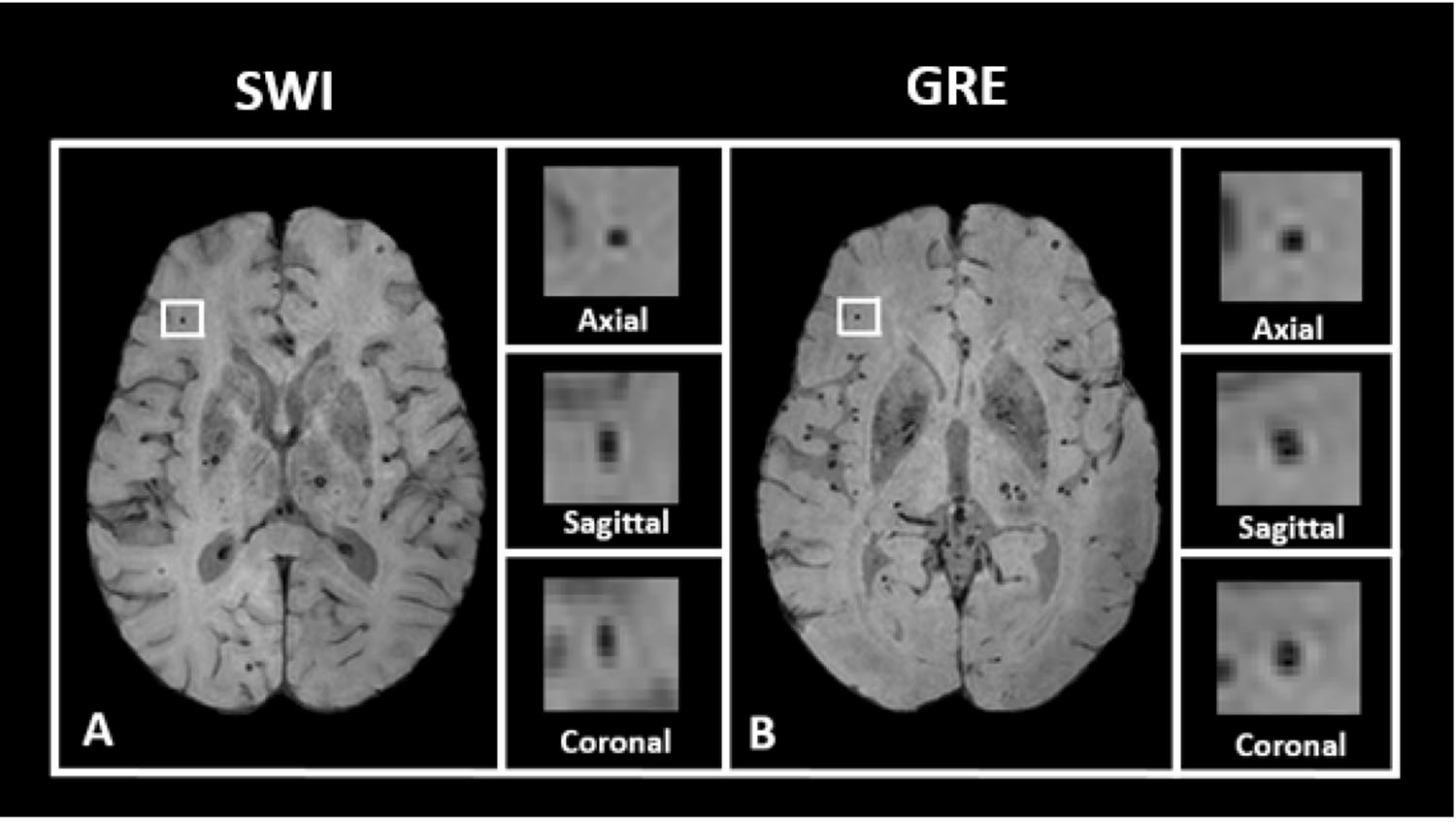
Examples of microbleed location. The participant’s MRI shown here reflects a heavy burden of cerebrovascular disease. (**A)** Left: axial slice of a SWI image, with a microbleed location highlighted in white. Right: an enlarged image of the highlighted location. From top to bottom, the views shown are axial, sagittal, and coronal. (**B)** Left: axial slice of the same participant’s GRE image, with the same microbleed location highlighted in white. Right: an enlarged image of the highlighted location. From top to bottom, the views shown are axial, sagittal, and coronal.

Microbleeds are detected radiologically with T2*-weighted MRI images, including either gradient echo (GRE) or susceptibility weighted images (SWI)^1–5,8–10^. These radiological findings have been validated with post-mortem analysis^6,7^, with true positives captured on imaging 48–89% of the time, depending on acquisition parameters^5^. Given the high level of sensitivity of MRI to paramagnetic material and the small size of the deposits, it is possible that MRI is more sensitive than gross pathological examination^4,10,11,23^. By omitting a refocusing pulse used in spin-echo sequences (such as T1) to correct of susceptibility distortion, GRE MRI is sensitive to paramagnetic artifacts, which can be exploited to visualize cerebral microbleeds^5,24^. SWI MRI is an alternative, more sensitive imaging modality for microbleed detection, with a larger “blooming” effect of paramagnetic material, making microbleeds more easily visible but also potentially more irregularly shaped^23,24^. Figure 1 shows an illustration of a microbleed using both SWI and GRE, highlighting the differences between the two modalities.

Visual inspection of the T2* MRI scans for small, ovoid, hypointense regions indicative of microbleeds is the most frequently used method of rating microbleeds. Several methods have been developed to improve interrater reliability and reduce the subjectivity inherent in visual reads^1,9,13^. With increased research and clinical interest in microbleeds, particularly with respect to ARIA-H, there is a need for standardized automated or semi-automated pipelines to detect cerebral microbleeds. A few methods have been proposed^14–17,25–31^. These studies, however, are frequently done in small^15,16,27^ clinical populations (e.g., patients with radiation-induced microbleeds^15–17^ or traumatic brain injury^14^), with much higher rates of microbleeds than in community-based adults and have not, to our knowledge, demonstrated generalizability to community-based samples, across MRI sequences, or with respect to the reliability of longitudinal detection. We present here the algorithm for Microbleed Automated detection using Geometric Identification Criteria (MAGIC), designed to detect and label cerebral microbleeds in predominantly healthy older adults. We selected participants from a community-based study rather than a clinical sample to test and illustrate our method. We demonstrate the high sensitivity and low false positive rate for the across both GRE and SWI images. This level of validation was not shown in other studies, to the best of our knowledge. We also demonstrate that our algorithm outperforms visual ratings in longitudinal stability of identifying microbleeds on SWI images and demonstrate that final visual inspection of the segmentation output by MAGIC significantly reduces the time needed to rate scans.

## Methods

### Participants

Participants were selected from the Washington Heights-Inwood Columbia Aging Project (WHICAP), a community-based study of cognitive aging and dementia among Medicare-eligible residents of northern Manhattan New York. WHICAP participants were recruited in 3 waves, beginning in 1992, 1999, and 2009. MRI was first introduced into WHICAP in 2004^32^ using a 1.5T MRI system and repeated on a subset of participants. Beginning in 2011, participants from the cohort recruited in 2009 received high-resolution MRI scanning using a 3T MRI system, and scans were once again repeated after 4.9 ± 1.3 (mean ± standard deviation) years on a subset of these participants. Randomly selected subsets of participants with available 3T MRI scans, including both SWI and GRE sequences, were included in this study (n = 78): one group (n = 44) was randomly selected from individuals rated visually as having at least one microbleed; the other group (n = 34) was randomly selected from individuals rated visually as not having any microbleeds. Fourteen of the microbleed positive participants had a follow-up MRI scan including SWI available at the time this study was performed, so these participants formed our longitudinal sample. GRE images were not collected at follow-up. The WHICAP study is approved by the Columbia University Medical Center Institutional Review Board, and all participants gave written informed consent.

### MRI acquisition

Magnetic resonance images were obtained using a 3T Philips Intera scanner at Columbia University between 2011 and 2018. T1-weighted (repetition time = 6.6 ms, echo time = 3.0 ms, field of view = 256 × 200mm^2^, 1-mm slice thickness), T2*-weighted SWI (repetition time = 17 ms, echo time = 24 ms, field of view = 244 × 197mm^2^, 2 mm slice thickness, in plane resolution 0.43 × 0.43 mm), and T2*-weighted GRE (repetition time = 15 ms, echo time = 22 ms, field of view = 220 × 181mm^2^, 1 mm slice thickness, in plane resolution 0.43 × 0.43 mm) Magnetic resonance images were acquired for each participant at baseline, and T1-weighted and T2*-weighted SWI images using the same parameters were acquired for the subset of participants who completed follow-up scans.

### Visual microbleed ratings

Consistent with previous studies done in the WHICAP cohort^3^, microbleeds were rated by visual inspection using criteria suggested by Greenberg and colleagues^1^. These criteria include the following guidelines: a dark (black) lesion on T2*-weighted MRI, accompanied by a “blooming” effect, which is round or ovoid and at least half-way surrounded by parenchyma (to distinguish microbleeds from vessels). The microbleed is devoid of signal hyperintensity on accompanying T1-weighted sequences and is distinguishable from other mimics (e.g., calcium deposits, bone, or vessel flow). Microbleeds were visually classified by location, including lobar (frontal, temporal, parietal, and occipital lobes) and deep (basal ganglia, thalamus, and infratentorial regions) locations. The number of microbleeds and location were noted for each participant. Three raters (IBM, EA, AGC), each trained in visually identifying microbleeds (IBM with over eight years of experience, AGC and EA with two years of experience), rated the entirety of the SWI and GRE scans, and microbleed locations, which were identified as true locations by either two or three raters, were used as the ground truth locations for testing the sensitivity of the algorithm, with unanimously identified locations representing definite microbleeds and locations identified by only two raters representing potential microbleeds. For longitudinal validation, two of the three raters rated the repeat SWI scans for microbleeds. In this study, participants were designated as microbleed positive if two or three raters agreed there was at least one microbleed in the brain, and microbleed negative if all raters agreed that no microbleeds were present. (Participants who had a microbleed identified by only one rater were excluded from this analysis. They would be considered microbleed negative by visual rating standards, but we wanted to maximize the difference between true and false positives, so we excluded these as too ambiguous.) Both percentage agreement (defined as the number of locations labeled by both raters divided by the total number of locations labeled) and Fleiss’ kappa^33,34^ were used to assess interrater and intra-rater reliability across modalities. These assessments were performed to ensure the visual ratings provided a reliable ground truth to judge the algorithm-segmented microbleeds against. While the kappa score is frequently used to assess interrater agreement, it applies a very conservative estimate of rater agreement by correcting for probability of agreement in pure guessing. The true agreement level typically lies somewhere between the uncorrected agreement and the kappa score, so we use both methods to assess agreement between visual ratings.

### MRI preprocessing

**Figure 2:**
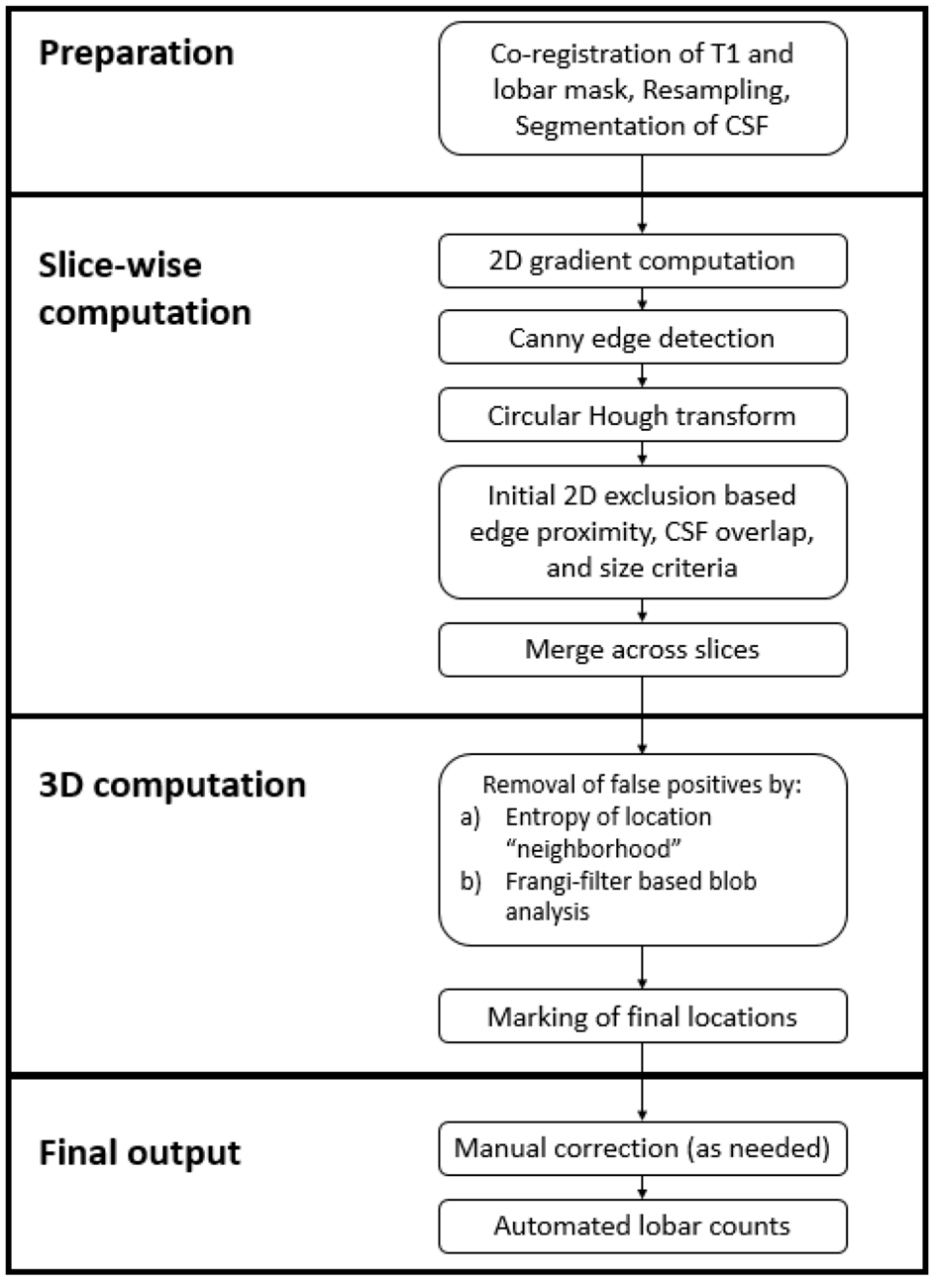
Algorithm flowchart. Summary of the algorithm as described in the text.

**Figure 3:**
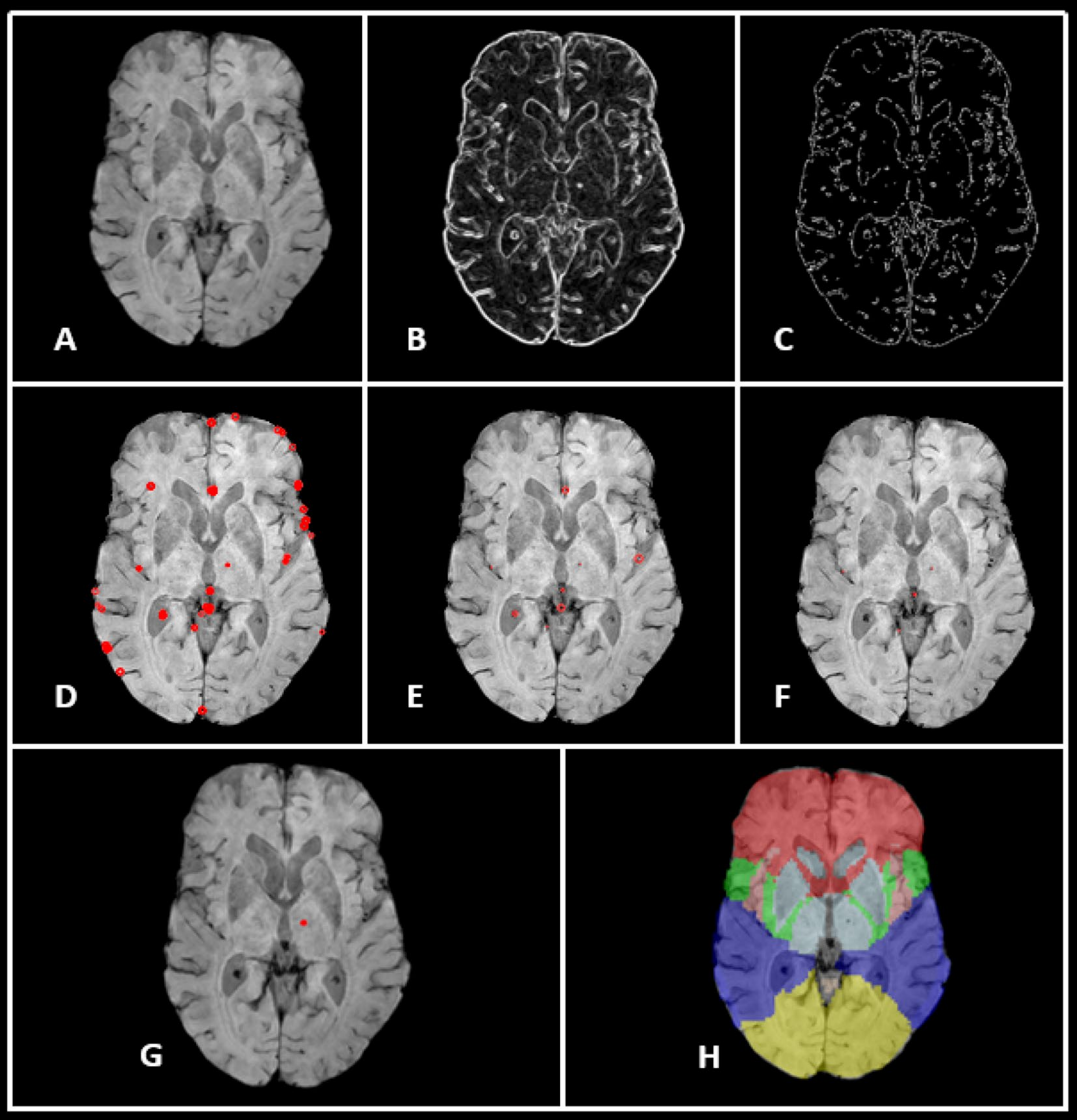
Algorithm Illustration. This image illustrates some of the algorithm steps on an SWI image. (**A)** Initial SWI image, resampled. (**B)** 2D gradient computation (gradient magnitude pictured). (**C)** Edges of the slice as output after Canny edge detection. (**D)** Initial potential ROIs labeled by the circular Hough transform. (**E)** ROIs remaining after edge exclusion. (**F)** ROIs remaining after circular grouping and size exclusion. (**G)** Final ROI marked after CSF exclusion and multi-slice merging. (**H)** Co-registered lobar map used to quantify distribution of detected microbleeds.

The entire algorithm pipeline is described by the flowchart in Fig. 2, and the steps are illustrated in Fig. 3. Before the microbleed detection could begin, a few preprocessing steps were required. The SWI and GRE scans for each participant were brain extracted using FSL Brain Extraction Toolbox^35^ (fsl.fmrib.ox.ac.uk/fsl/). The T1-weighted image and a lobar mask from FSL’s MNI atlas were co-registered to the SWI and GRE images separately; identification of microbleeds was done in the native space of each SWI and GRE scan. The co-registered T1-weighted volume was used to compute the CSF mask using the Statistical Parametric Mapping toolbox^36^ (SPM 12; www.fil.ion.ucl.ac.uk/spm/software/spm12/). Finally, the SWI and GRE scans were resampled to a higher resolution (Fig. 3A) so that all artifacts that were potentially microbleeds had a diameter of at least six voxels to ensure an accurate identification when using a circular Hough transform (see below). In the current implementation, this step was accomplished by scaling the images by a factor of three.

### Detection of potential microbleed regions of interest

Initially, potential microbleed locations were identified as circular regions of interest (ROIs) on each slice. For each slice of the GRE or SWI image, the 2D image gradient was computed with a 3 × 3 Sobel filter^37^ (Fig. 3B). Then, edge pixels were detected using the Canny edge detection algorithm^38^ to remove all neighboring voxels that are not local maxima. Hysteresis thresholding (lower bound = 0.1, upper bound = 0.15) was used to remove spurious edges as a result of noise. The final edges left after this method are illustrated in Fig. 3C.

Once the edge pixels were identified, a circular Hough transform^39^ was used to detect circular ROIs on each slice. We chose the circular Hough transform to identify these ROIs over other methods (notably over the radial symmetry transform, which has been suggested as a method of detecting microbleeds previously^15,26,40^) because it allows for a more lenient definition of circularity, and it is therefore more sensitive to ovoid shapes^41^. Potential circular ROIs on each slice were identified after restricting the radius to vary within a physiologically useful range^1,9^ ($r\in [5, 12]$, 0.72–1.72 mm) and thresholding at a lenient threshold of 80% of the maximum overlap in the Hough transform (Fig. 3D).

The large number of potential locations was then thinned using physiologically relevant criteria, analogous to the criteria used in visual inspection. First, all ROIs lying on the edges of the image were discarded. Overlapping ROIs that remain were merged together, and any that were too large to be true microbleeds, using a lenient cutoff of distance between centers greater than eight pixels (1.15 mm), or singular ROIs (i.e., circles unmerged with others, indicating an edge arising from noise) were excluded (Fig. 3F). Finally, all ROIs that overlapped with the CSF mask (segmented from co-registered T1) were excluded as vessels, similar to the visual rating criteria. The remaining locations marked on each slice were then merged across slices. The final ROI representing a potential microbleed was defined as the 3D center of the potential microbleed and a surrounding neighborhood of a standardized size (51 × 51 × 25 voxels, or two times the maximum expected size of a microbleed). This definition was used because a neighborhood of this size both ensures the entire microbleed artifact will be captured for analysis in the next stage and also standardizes the selected ROIs, making geometric features more comparable. At this stage of the algorithm, we tested the sensitivity of detection of microbleeds compared with ground truth visual ratings to ensure that the automatic labelling was accurately capturing the visually labelled microbleeds.

### 3D geometric filtering

To assist in the removal of false positive locations, we used the geometric information contained in each ROI identified in the previous step. We selected a priori four characteristics of the ROI as having the potential to differentiate between true and false positive locations: the 3D image entropy of the ROI, the 2D image entropy of the maximum intensity projection of the ROI, and the volume and compactness of the central blob in each ROI as identified via Frangi filtering.

In an image, each pixel $i$ has a probability ${p}_{i}$ of being a given intensity, measured as the fraction of all pixels in the image at that intensity. Image entropy $E$ is defined^42^ based on this intensity probability distribution such that

1 $$E= -\sum_{i}{p}_{i}{\mathrm{log}}_{2}{p}_{i}$$

**Figure 4:**
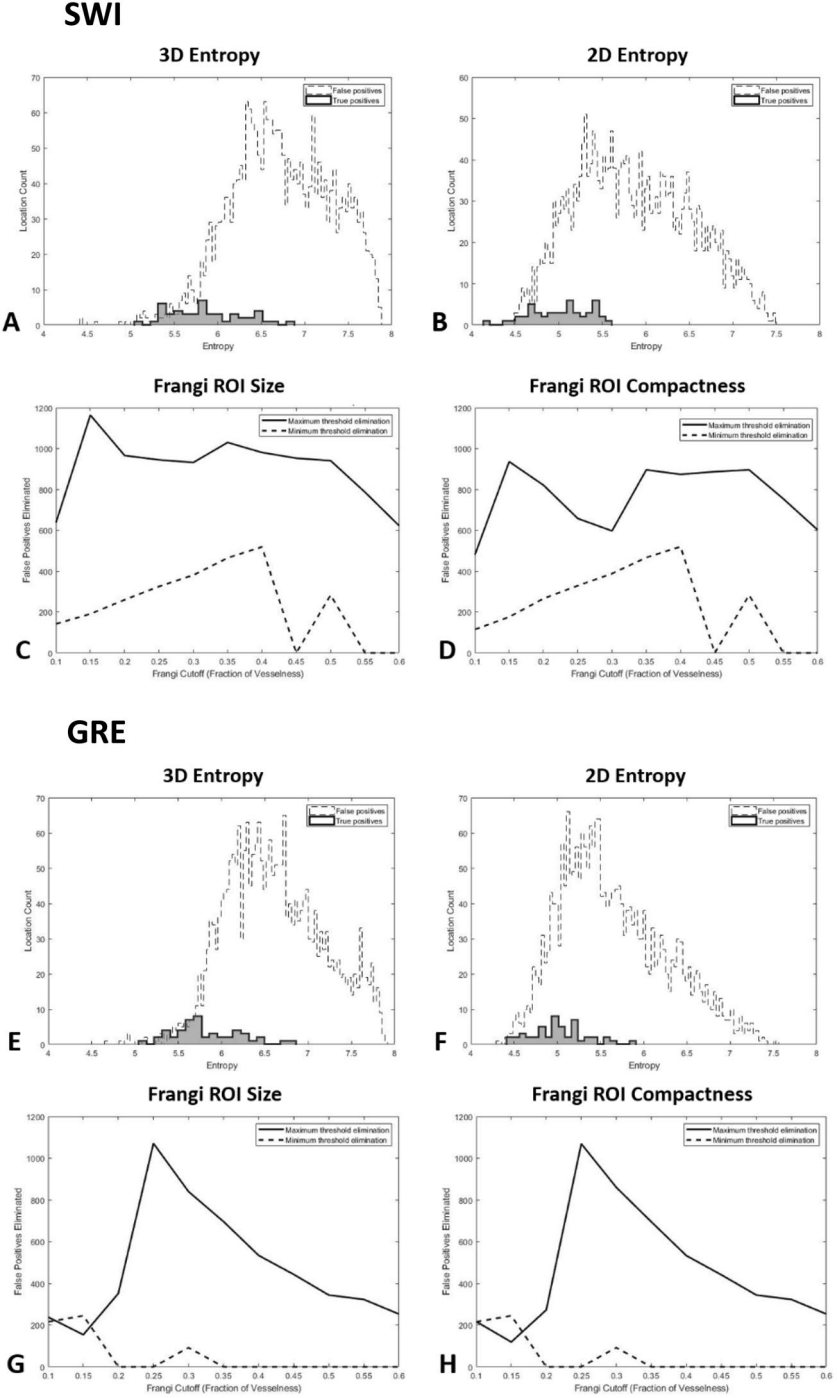
Geometric measure cutoff justifications. This figure illustrates the geometric properties used to remove false positives from the identified locations in SWI and GRE images. Modalities are separated into panels. (**A)** Distribution of 3D entropy in the ROI neighborhood on SWI. (**B)** Distribution of the 2D entropy of the maximum intensity projection of the ROI neighborhood on SWI. (**C)** Number of false positives removed at different cutoffs of the Frangi vesselness measure based on the central blob volume on SWI. (**D)** Number of false positives removed at different cutoffs of the Frangi vesselness measure based on the central blob compactness on SWI. (**E)** Distribution of 3D entropy in the ROI neighborhood on GRE. (**F)** Distribution of the 2D entropy of the maximum intensity projection of the ROI neighborhood on GRE. (**G)** Number of false positives removed at different cutoffs of the Frangi vesselness measure based on the central blob volume on GRE. (**H)** Number of false positives removed at different cutoffs of the Frangi vesselness measure based on the central blob compactness on GRE.

In a typical 8-bit greyscale image, entropy will lie in the range from zero (all pixels are the same intensity) to eight (all 2^8^ shades of grey have an equal chance of occurring). In a 3D image with a large signal void in the center surrounded by parenchyma, characteristic of a true microbleed, we expected a moderate amount of entropy, while in an area characterized by many sharp gradient changes, characteristic of a false positive, we expected a higher amount of entropy indicating a noisy, false positive region. In a 2D maximum intensity projection of a true microbleed, we expected a lower entropy than in the case of a false positive, as the sharp gradient change around a microbleed tends to leave a small hyperintense ring around the location. However, as this feature is much smaller than the signal void, we did not expect the 2D entropy to be as sharply distinctive as the 3D entropy. True and false positive ROI entropies are illustrated in Fig. 4.

The Frangi filter^43^ utilizes the second order derivatives of an image to extract spatial information about the geometry of an ROI. In a 3D image $I$, the Hessian matrix $H$ at each location is defined as the matrix

2 $$H=\left[\begin{array}{ccc}{I}_{xx}& {I}_{xy}& {I}_{xz}\\ {I}_{yx}& {I}_{yy}& {I}_{yz}\\ {I}_{zx}& {I}_{zy}& {I}_{zz}\end{array}\right]$$

The eigenvalues of the Hessian ${\lambda }_{1}, {\lambda }_{2},{\lambda }_{3}$ are defined to be ordered such that $\left|{\lambda }_{1}\right|\le \left|{\lambda }_{2}\right|\le \left|{\lambda }_{3}\right|$. When the structure of interest is hypointense compared with the surroundings, which is true for both microbleeds and vessels on T2*-weighted images, ${\lambda }_{1},{\lambda }_{2},{\lambda }_{3}\ge 0$. The vesselness of each voxel $i$ can therefore be described as

3 $$V\left(i\right)= \left\{\begin{array}{cc}0& \text{if }{\lambda }_{2}<0 \, \text{or }{\lambda }_{3}<0\\ \left(1-{\text{exp}}\left(-\frac{{R}_{A}^{2}}{{2a}^{2}}\right)\right){\text{exp}}\left(-\frac{{R}_{B}^{2}}{{2\beta }^{2}}\right)\left(1-{\text{exp}}\left(-\frac{{S}^{2}}{{2c}^{2}}\right)\right)& \text{in all other cases}\end{array}\right. $$

where ${R}_{A}$, ${R}_{B}$, and $S$ are ratios containing structural information from the eigenvalues, defined as

4 $${R}_{A}=\frac{\left|{\lambda }_{2}\right|}{\left|{\lambda }_{3}\right|}$$

5 $${R}_{B}=\frac{\left|{\lambda }_{1}\right|}{\sqrt{\left|{\lambda }_{2}{\lambda }_{3}\right|}}$$

6 $$S=\sqrt{{\lambda }_{1}^{2}+{\lambda }_{2}^{2}+{\lambda }_{3}^{2}}$$

and $\alpha ,\beta ,c$ are constants used to tune the sensitivity of the filter to the structural ratios. In our implementation, we used the suggested^43^ values of $\alpha =0.5, \beta =0.5$ and $c$ as half of the Hessian norm. The vesselness of an ROI can assist in separating true positive from false positive locations, since a tubular artifact, such as a vessel, will have a high vesselness within the region (${R}_{A} \sim 1$ and ${R}_{B}\sim 0$), an ovoid artifact will have a lower degree of vesselness (${R}_{A} \sim 1$ and ${R}_{B}\sim 1$), and an ROI containing high-gradient noise will have a vesselness approaching zero ($\left|{\lambda }_{1}\right|\sim \left|{\lambda }_{2}\right|\sim \left|{\lambda }_{3}\right|\sim 0$).

In a standard Frangi filter, when filtering for large tubular structures such as vessels, it is necessary to compute vesselness across a range of scales determined by different Gaussian filters. Since the artifacts we measured are relatively small and did not vary greatly in size, the benefits of maximizing over a range of scales was not worth the computational cost, so we used only the vesselness as measured in the resampled space without any Gaussian blur.

Once we computed the vesselness of all the voxels within each ROI, we used blob analysis to extract the central blob of each ROI defined as all non-zero voxels grouped via 26-connected neighborhood to the non-zero voxel closest to the center of the ROI. First, the vesselness within an ROI was used to create a binary mask, by thresholding as a fraction of the maximum vesselness within the ROI. We tested in a range of 0.1 (not zero to exclude noise) to 0.6 (higher levels are useful to distinguish vessels, not ovoid locations such as microbleeds). The volume, defined as the number of voxels with the blob, and compactness, defined as square of the number of perimeter voxels of the blob divided by the volume of the blob, were noted for this central blob. The number of false positives eliminated at this step using the volume and compactness (minimum and maximum cutoffs) are illustrated in Fig. 4. After this step, we also computed the precision of the algorithm (percentage of true positives to total number of labeled locations).

### Final counting and location step

After the vast majority of false positives were removed by the previous step, the remaining ROIs were saved in the native space of the modality of interest (SWI or GRE) in an easily viewable and editable format for correction by a trained rater. The difference in rating times between visual ratings and rating the automatically segmented images was evaluated in a separate group of 20 SWI scans. The co-registered lobar mask was used to count automatically the number of microbleeds identified and output the distribution of locations throughout the brain for further analysis. For the longitudinal scans, an additional visual rating was done using the algorithm’s output locations to confirm that the locations detected at multiple timepoints were indeed microbleeds (i.e. visual ratings were done blinded to the algorithm, and then redone using the algorithm’s output across both timepoints). We emphasize this step so as to be clear that visual ratings remain our gold standard method of rating microbleeds, even when they are informed by an algorithm.

## Results

### Demographic information

The demographic characteristics of the study cohort are presented in Table 1. Participants were designated as either microbleed positive (one or more microbleeds identified by two or more raters) or microbleed negative (no microbleeds identified by any rater). Microbleed positive participants were slightly older than microbleed negative participants (t(76) = 2.21, p = 0.03), but did not differ in terms of sex/gender (χ^2^(1, N = 78) = 1.37, p = 0.24) and race/ethnicity (χ^2^(3, N = 78) = 7.29, p = 0.06). The microbleed positive participants who had a follow-up MRI scan about five years later had similar distribution of sex/gender (χ^2^(1, N = 58) = 0.58, p = 0.45) and race/ethnicity (χ^2^(3, N = 58) = 2.09, p = 0.55) as the baseline sample of microbleed positive participants.

**Table 1:** Demographic characteristics of individuals with and without microbleeds.

Microbleed status	Positive	Negative	Total	Statistic
**Baseline**				
N	44	34	78	–
Age, years: mean (SD)	76.3 (6.0)	73.3 (7.0)	74.5 (6.6)	t = 2.21, p = 0.03
Sex/gender, women: N (%)	20 (45)	21 (60)	41 (52)	χ^2^ = 1.37, p = 0.24
Race/ethnicity: N (%)				χ^2^ = 7.29, p = 0.06
White	23 (52)	11 (32)	34 (44)	
Black	15 (34)	11 (32)	26 (33)	
Hispanic	4 (9)	11 (32)	15 (19)	
Other	2 (5)	1 (3)	3 (4)	
**Follow up**				
N	14	0	14	
Age, years: mean (SD)	79.3 (6.1)	–	79.3 (6.1)	
Time to follow-up, years: mean (SD)	4.86 (1.3)	–	4.86 (1.3)	
Sex/gender, women: N (%)	8 (57)	–	8 (57)	
Race/ethnicity: N (%)		–		
White	5 (36)	–	5 (36)	
Black	5 (36)	–	5 (36)	
Hispanic	3 (21)	–	3 (21)	
Other	1 (7)	–	1 (7)	

Microbleed positive participants are those who have at least one microbleed present (identified by two or more raters), and microbleed negative participants are those who have no microbleeds present (agreed by all three raters).

### Interrater reliability

A potential microbleed was labeled as a “definite” microbleed if all three raters agreed that the artifact was a microbleed, and as a “probable” microbleed if two raters agreed that it was a microbleed (full rating agreement can be found in Supplementary Table S1). There was an acceptable level of agreement between raters, with agreement ranging from 0.67 to 0.97 depending on rater and imaging modality. Interrater reliability did not differ systematically between SWI (0.67–0.95) and GRE (0.67–0.97). Merged ratings, reflecting the combination of SWI and GRE ratings via OR operation (i.e., if a rater labeled the location as a true positive on either SWI or GRE they counted it as a true microbleed) were also computed, and showed similar agreement range (0.70–0.95). Interrater reliability, measured across both microbleed positive and negative participants, was similar across modalities (SWI: κ = 0.714, 95% CI: [0.710, 0.717]; GRE: κ = 0.708, 95% CI: [0.705, 0.712]; merged: κ = 0.733, 95% CI: [0.729, 0.737]) and comparable to prior studies that used visual ratings^1^. Intra-rater reliability between modalities was similar (Rater 1: κ = 1.00, 95% CI: [0.994, 1.006]; Rater 2: κ = 0.751, 95% CI: [0.745, 0.758]; Rater 3: κ = 0.726, 95% CI: [0.720, 0.733]).

Visual ratings identified 54 locations across the 44 microbleed positive participants using SWI scans (39 definite locations, 15 probable locations). In the same 44 participants, visual ratings identified 61 locations on GRE (43 definite locations, 18 probable locations). Combining these ratings, there were a total of 64 unique locations identified (45 definite locations, 19 probable locations). These visual results were used as the “ground truth” measure of sensitivity for the algorithm.

### Algorithm results—sensitivity

Of the 54 locations found on SWI, the algorithm identified 50 (38 of the definite true positives, 12 of probable true positives, 93% overall sensitivity). Of the 61 locations found on GRE, the algorithm identified 56 (41 of the definite true positives, 15 of the probable true positives, 92% overall sensitivity). Combining the ratings, the algorithm identified 61 true locations (44 of the definite true positives, 17 of the probable true positives, 95% overall sensitivity). Treated as an independent rater, the algorithm achieved a high level of agreement with other raters in marking true microbleed locations (0.75–0.89), higher than the average agreement amongst visual ratings. The full results of the algorithm’s sensitivity are shown in Table 2.

**Table 2:** Algorithm sensitivity results.

	Definite	Probable	Combined
**SWI**			
Rater identified	39	15	54
Algorithm identified	38	12	50
Sensitivity	0.97	0.8	0.93
**GRE**			
Rater identified	43	18	61
Algorithm identified	41	15	56
Sensitivity	0.95	0.83	0.92
**Merged**			
Rater identified	45	19	64
Algorithm identified	44	17	61
Sensitivity	0.98	0.89	0.95

An artifact was labeled as a “definite” microbleed if all three raters agreed that the artifact was a microbleed, and as a “probable” microbleed if two raters agreed that it was a microbleed. This table shows the sensitivity results of the algorithm across these different labels. Note that in a study using only visual ratings, the final column (combining the definite and probable ratings) would be the number typically reported.

### Algorithm results—precision

After removing false positives using the cutoff criteria derived from the geometric measures, the algorithm identified an average of 9.7 false positives per scan (precision: 11%) on SWI images and an average of 17.1 false positives per scan (precision: 7%) on GRE images. The performance on microbleed negative participants was modestly better, with an average of 7.32 and 15.4 false positives per scan on SWI and GRE, respectively. The full results of the algorithm’s precision are shown in Table 3.

**Table 3:** Algorithm precision results.

	True positives	False positives	Precision	Average FP/scan
**Microbleed positive**				
SWI	50	426	0.11	9.7
GRE	56	752	0.07	17.1
**Microbleed negative**				
SWI	–	249	–	7.32
GRE	–	544	–	15.4
**Follow up**				
SWI	10	160	0.06	3.64

As noted in the methods, microbleed positive participants are those who have at least one microbleed present (identified by two or more raters), and microbleed negative participants are those who have no microbleeds present (agreed by all three raters). False positive (FP) are presented in the final column averaged over the number of images (FP/scan).

The measures of 3D entropy in true positive locations did not differ between SWI (average entropy: 5.85 ± 0.41; entropy range 5.06–6.88) and GRE (average entropy: 5.79 ± 0.38; entropy range: 5.07–6.88). As we hypothesized, the 3D entropy of false positives was (Supplementary Tables S2A and S3A) higher than the distribution of true positive locations in both SWI (average entropy: 6.74 ± 0.58, p < 0.001) and GRE (average entropy: 6.63 ± 0.57, p < 0.001) images. In parallel with these results, the 2D entropy of the maximum intensity projections was lower (Supplementary Tables S2A and S3A) in true positive locations (SWI average entropy: 5.01 ± 0.33; GRE average entropy: 5.01 ± 0.31) than in false positive locations (SWI average entropy: 5.87 ± 0.66, p < 0.001; GRE average entropy: 5.67 ± 0.62, p < 0.001). In SWI images, the 2D entropy provided a more sensitive discriminant between true and false positives (2D eliminates 35.9 false positives per scan, 3D eliminates 24.5 false positives per scan), while in GRE the 2D and 3D entropy provided roughly the same level of discrimination (19.8 and 19.9 false positives per scan eliminated by 2D and 3D, respectively). Nearly all of the eliminated false positives had an entropy higher than the range of true positives, with the few that fell below the range lying in locations near a larger signal void (e.g., infarct) that would be visually rated as too large to be a microbleed. The relative distributions of 3D and 2D entropy are shown illustrated in Fig. 4 (Fig. 4A,B show 3D and 2D entropy, respectively, in SWI, with Fig. 4E,F illustrating the same in GRE).

As we expected, lower values of the Frangi filter cutoff allowed for generally better discrimination between true and false positives likely due to the relatively low vesselness of the structures measured. At the cutoffs selected to maximize difference between false positives larger than true microbleeds (SWI: 0.15; GRE: 0.25; see Tables S2B and S3B for cutoffs used for ROIs smaller than true microbleeds as well), true positive volume was lower on average than the volume of false positives on both SWI (true positive (voxels): 1245 ± 475; false positive: 3003 ± 1794; p < 0.001) and GRE (true positive: 54 ± 414; false positive: 2060 ± 1287; p < 0.001). Volume cutoffs were useful in eliminating several false positives, on both SWI (minimum: 11.8 false positives per scan eliminated; maximum: 26.5 false positives per scan eliminated) and GRE (minimum: 1.4 false positives per scan; maximum: 24.3 false positives per scan eliminated). Supplementary Tables S2B and S3B provide the full results for different volume cutoffs in SWI and GRE, respectively. In parallel with these results, compactness was lower in true positives (SWI: 540 ± 414; GRE: 456 ± 309) than in false positives (SWI: 2060 ± 1287, p < 0.001; GRE: 884 ± 697, p < 0.001; see Supplementary Tables S2C and S3C for full results). Because the extreme volume difference between true positive and false positive results drove this relationship, compactness did not provide greater discrimination ability beyond volume, contrary to our initial hypothesis. These results are illustrated for Fig. 4 (Fig. 4C,D illustrate ROI size and compactness, respectively, in SWI, with 4G and 4H illustrating the same measures in GRE).

**Figure 5:**
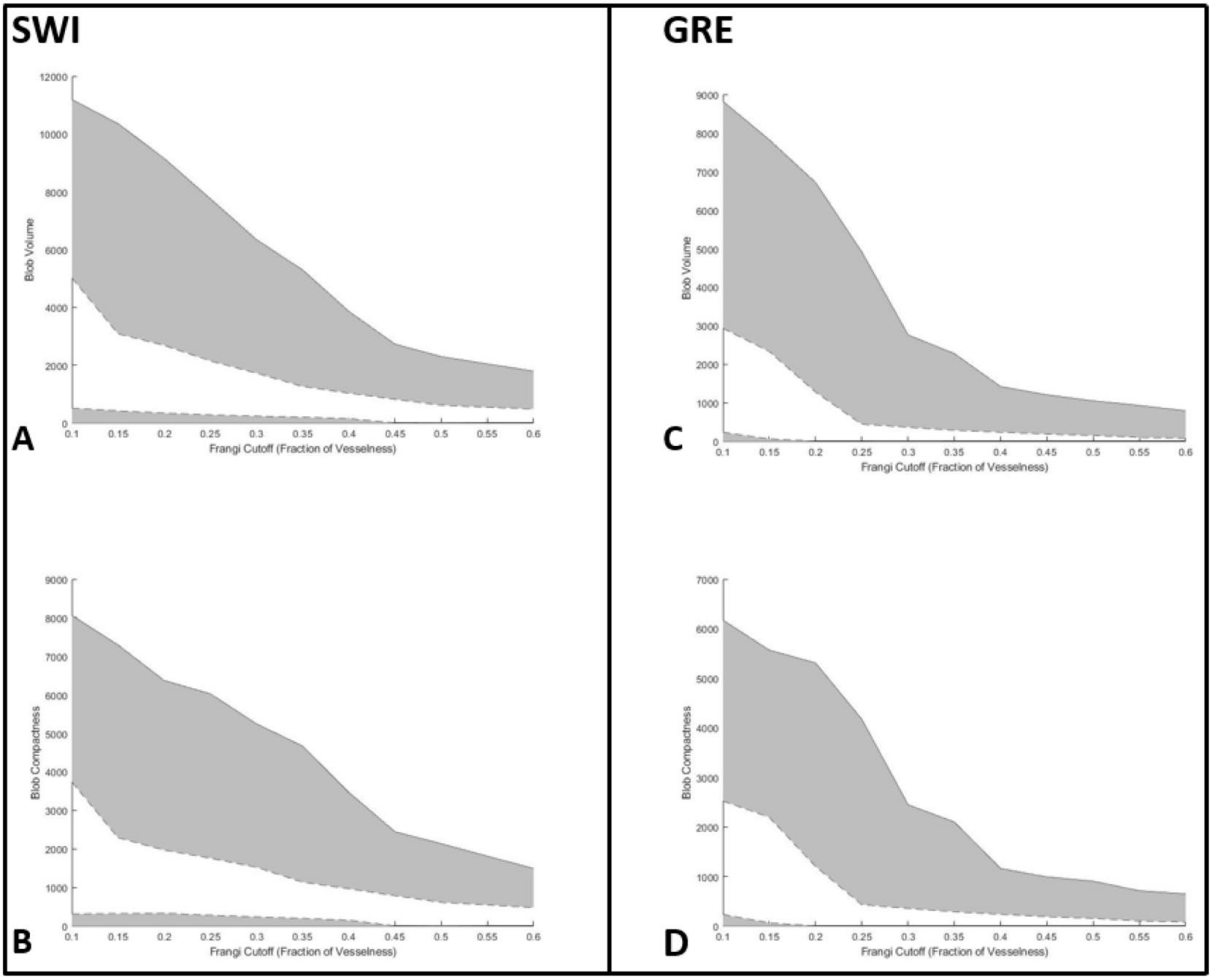
Illustration of Frangi-filter threshold effect on blob size and compactness. The dashed lines represent the bounds of the volumes of true positives, while the solid lines represent the bounds of the volumes of false positives. All false positives that fall within the shaded grey areas are removed. The purpose of trying a range of thresholds for vesselness was to determine the points of maximum difference (i.e., where the shaded area is largest, and therefore removes the most false positives). (**A)** Range of volume of central blobs in Frangi-filtered ROIs on SWI images across different thresholds. (**B)** Range of central blob compactness in Frangi-filtered ROIs on SWI images across different thresholds. (**C)** Range of volume of central blobs in Frangi-filtered ROIs on GRE images across different thresholds. (**D)** Range of compactness of central blobs in Frangi-filtered ROIs on GRE images across different thresholds.

Figure 5 presents a visual summary of how volume and compactness change across different values of Frangi filter cutoffs. The top row (Fig. 5A,C, illustrating SWI and GRE, respectively) demonstrates that while true positive volume was lower than most false positive volumes (the shaded grey region), these differences were heightened in lower cutoffs for the Frangi filter, indicating that these values provide a greater level of discrimination between true and false positive locations. The bottom row (Fig. 5B,D, again SWI and GRE, respectively) show the same pattern in compactness across Frangi filter cutoff. The cutoff values were chosen to maximize the difference between true and false positives (the shaded grey area), providing the greatest level of precision.

It is interesting to note that for the maximum cutoffs, which are responsible for more false positive eliminations than the minimum cutoffs, a cutoff of 0.25 could be used on both SWI and GRE to simplify implementation. Although we did not use this cutoff, as the cutoff would slightly lower the precision because it allows for more false positives to remain, it could provide a simpler form of analysis.

### Final counting and location step

A random sample of 20 SWI scans from WHICAP (different from the ones used to develop the algorithm) was used to test the speed of visual ratings versus the editing of locations identified by the algorithm. The time to rate a group of 10 scans visually (no algorithm masks) was 6.12 ± 1.58 min (mean ± standard deviation). The time to rate a group of 10 scans with the algorithm mask was 3.48 ± 1.81 min, significantly (t(17.6) = 3.50, p = 0.003) reducing the time to rate scans by 43%.

Nearly all (92%) of the microbleeds in this sample occurred in lobar locations (frontal: 42%, temporal: 18%, parietal: 26%, and occipital 6%). Microbleeds lying within deeper brain structures accounted for the remainder (basal ganglia: 4%, cerebellum: 4%).

### Longitudinal results

In the 14 participants who had longitudinal scans available, there were 20 potential true microbleed locations identified at baseline. In the longitudinal scans, visual ratings identified a subset of these locations remaining (rater #2 identified 6 locations; rater #3 identified 5 locations), while the algorithm identified 10 of the original locations on the follow-up scans, greatly outperforming the visual ratings in terms of longitudinal reliability. Applying the cutoffs defined at baseline to the longitudinal dataset did not remove any of the true positives and resulted in a similar level of precision to the baseline SWI (3.6 false positives per scan; 5.9% precision), indicating that the cutoffs derived using only the baseline data were applicable across multiple timepoints.

## Discussion

We have presented here MAGIC, a novel automated method to detect cerebral microbleeds on images acquired at 3T field strength. This new method has high sensitivity and reasonable precision on both T2*-weighted GRE and SWI images, both of which are commonly used in research and clinical applications. We validated the method in a community-based cohort, using both microbleed positive and negative participants. The automation we present, even with its need for minor manual corrections of false positives (a visual inspection step required to ensure the accuracy of the final results), reduces the time needed to visually rate the scan by drawing the rater’s attention to areas most likely to be microbleeds, while retaining a high sensitivity to the microbleeds themselves. We also demonstrated, in a small subset of participants, that the automated algorithm exhibits higher sensitivity in longitudinal identification of potential microbleed locations than visual ratings. Longitudinal reliability in identifying persistent artifacts is critical to future assessments of microbleeds, as the longitudinal stability of microbleed artifacts remains unknown, and accurate identification on multiple scans would help to elucidate the degree to which they change in appearance over time^23,44^.

As we expected, SWI provides a greater level of precision in identifying microbleeds compared to GRE. This observation is due to the underlying nature of the sequences, as GRE is able to show paramagnetic artifacts, while SWI provides a more sensitive measure by incorporating phase information into the image itself^1,2,5,10^. Although these differences exist, the strength of the algorithm we present is that it works with comparable sensitivity and precision on both SWI and GRE scans, allowing for use across many different clinical and research applications.

Aside from the demonstrated ability of MAGIC to work across multiple modalities as well as longitudinal identification of microbleeds, features that have not been shown in any other algorithm to our knowledge, we believe that the interpretability of all our steps to provide an attractive additional feature. In comparison to other proposed solutions that sacrifice interpretability (e.g., machine-learning based approaches, such as convolutional neural networks^16,25,31^, which do not offer an interpretable set of features), the simple geometric measures we propose correspond well with the criteria used for visual rating^1^, and the cutoff values can be easily modified to accommodate different acquisition parameters used by different groups. Additionally, we do not need to compute a high dimensional set of geometric features and select by weight, as proposed in certain random forest implementations^14,29,30^. By maintaining interpretable geometric methods throughout the algorithm, we believe it is easier to adapt the pipeline to study or scanner specific differences on the basis of a few experimental scans, rather than requiring retraining for each site or training on a very large initial sample set.

As with any algorithm, there are always limitations and room for optimization. When compared to other automated or semi-automated pipelines already in existence, the method we propose here achieved a higher sensitivity^15,17,26,31^ and precision^14,27^ compared to the majority of existing methods. There are some distinct advantages to other methods, however, which must be acknowledged. Deep convolutional networks, while requiring more initial processing and manual labeling, can leverage large datasets to achieve a significantly higher precision than purely geometric analyses^25,30,31^, as they are able to better discriminate between edge artifacts and true positives (geometric criteria are susceptible to confusion in sharp gradients that higher dimensional approaches, such as convolutional networks or statistical shape maps, are able to resolve). Another approach that has promise^29^, particularly when it is not feasible to acquire higher resolution scans, combines information from SWI images and proton-density weighted images to verify the accuracy of the microbleed locations and remove fluid mimics (e.g. vessels). To our knowledge, no other automated method has addressed longitudinal reliability, so the data we provide here are the first of their kind. Additionally, we did not find any explicit comparison of method reliability across SWI and GRE scans.

There are certain approaches in currently existing methods, such as combining multiple modalities^29^, or extracting information from the area surrounding an ROI as well as the ROI itself^25,30,31^, which could be leveraged to increase the precision of our approach. One way of improving the algorithm without additional image collection is to explicitly include phase information from the SWI images, as SWI is more sensitive to paramagnetic deposits because it incorporates the distortions within the phase image into the scan^23^. We would look to explicitly process the phase image as well as the final SWI image to look for these specific local distortions as a way of reducing the number of false positives due to gradient changes unrelated to susceptibility effects (e.g., cerebellar folds). Additionally, the inclusion of phase image information would allow for a more specific determination of iron deposition, especially when compared to calcium deposits, as they shift the phase in opposite directions^5^. We are also considering refinements in our definition of entropy, such as mutual information entropy (i.e., weighting local clusters of voxels by how similar their entropy is, rather than computing entropy solely on an entire image) as a way to further tune the sensitivity of this measure to the presence of true positives. However, given the promising results we already show with this version of the algorithm, we believe these changes will provide incremental benefits. As high-quality imaging becomes more common, especially in the evolving area of microbleed analysis, we believe that algorithmic identification of microbleeds will become a greater necessity, and MAGIC presents a promising step towards achieving a broadly applicable, automated approach to microbleed identification.

Considering the observed association between microbleeds and diseases such as cerebrovascular disease^2,3,10–12^, cerebral amyloid angiopathy and AD^1,4,19,20^, as well as the critical role microbleeds may play in treatment trials^4,21,22^, we believe that a standardized way to identify microbleeds, cross-sectionally and longitudinally, that is generalizable across cohorts will become imperative in assessing microbleed burden. As the elderly population continues to grow, creating consistent, broadly applicable ways of quantifying microhemorrhage location and burden will become increasingly important in both ensuring a high standard of clinical care and providing reliable data to uncover biological causes of microbleeds and how they relate to these diseases.

## Supplementary Information

Supplementary Tables.

## Data Availability

Qualified investigators may request data in writing (see http://www.cumc.columbia.edu/adrc/investigators for more details).
