# Supplementary material for: Automated detection of cerebral microbleeds on T2*-weighted MRI
Source: Sci Rep. 2021 Feb 17;11:4004. doi: 10.1038/s41598-021-83607-0 (PMC7889861; doi:10.1038/s41598-021-83607-0)
Supplement: Supplementary file 1 — Supplementary Tables. [file 41598_2021_83607_MOESM1_ESM.docx]

**Automated detection of cerebral microbleeds on T2*-weighted MRI**

Anthony G. Chesebro, BS,^1^ Erica Amarante, BA,^1^ Patrick J. Lao, PhD^1^, Irene B. Meier, PhD,^1^ Richard Mayeux, MD,^1,2,3^ and Adam M. Brickman, PhD^*1, 2, 3^

1. Taub Institute for Research on Alzheimer’s Disease and the Aging Brain, College of Physicians and Surgeons, Columbia University, New York, NY, USA.
2. Gertrude H. Sergievsky Center, College of Physicians and Surgeons, Columbia University, New York, NY, USA.
3. Department of Neurology, College of Physicians and Surgeons, Columbia University, New York, NY, USA.

^*^Corresponding author

Adam M. Brickman, Ph.D.

Taub Institute for Research on Alzheimer’s Disease & the Aging Brain

Department of Neurology

College of Physicians and Surgeons

Columbia University

630 West 168^th^ Street

PS Box 16

New York, NY 10032

Tel: +1 212 342 1348

Fax: +1 212 342 1838

Email: [amb2139@columbia.edu](mailto:amb2139@columbia.edu)

**Supplementary Information**

Table S1 provides the full interrater agreement comparisons summarized in the second section of the Results. Tables S2 and S3 provide the data used to set the entropy cutoffs, Frangi filter thresholds, and filtered blob size and compactness cutoffs along with statistical tests to determine the differences in measures in true and false positive locations.

Table S1 – Interrater Agreement

Table S2 – Entropy and Frangi-filter cutoff derivations for SWI images

Table S3 – Entropy and Frangi-filter cutoff derivations for GRE images

**Supplementary Table S1. Interrater Agreement.** These statistics reflect the percentage agreement between raters across modalities. Percentage agreement is defined as the number of locations identified by both raters divided by the total number of locations identified. Merged ratings reflect the combination of SWI and GRE ratings via OR operation (i.e., if a rater labeled the location as a true positive on either SWI or GRE they counted it as a true microbleed).

| **SWI** |  |  |  |
| --- | --- | --- | --- |
|  | Rater 1 | Rater 2 | Rater 3 |
| Rater 1 | - | 0.67 | 0.72 |
| Rater 2 | 0.67 | - | 0.95 |
| Rater 3 | 0.72 | 0.95 | - |
| **GRE** |  |  |  |
|  | Rater 1 | Rater 2 | Rater 3 |
| Rater 1 | - | 0.67 | 0.70 |
| Rater 2 | 0.67 | - | 0.97 |
| Rater 3 | 0.70 | 0.97 | - |
| **Merged** |  |  |  |
|  | Rater 1 | Rater 2 | Rater 3 |
| Rater 1 | - | 0.70 | 0.75 |
| Rater 2 | 0.70 | - | 0.95 |
| Rater 3 | 0.75 | 0.95 | - |

**Supplementary Table S2. A.** Entropy cutoffs and distributions for SWI images across true positives (TP) and false positives (FP). **B.** Volume of central blobs in Frangi-filtered ROIs on SWI images across different thresholds. Note that the cutoffs chosen were based on the distributions that gave the maximum difference between true positives and false positives. **C.** Compactness of central blobs in Frangi-filtered ROIs on SWI images across different thresholds. (*marks the threshold values selected, 0.15 for max cutoff, 0.4 for min cutoff)

|  |  | | 1. **SWI Entropy** | |  | |  |  |
| --- | --- | --- | --- | --- | --- | --- | --- | --- |
|  | **False Positives Eliminated** | | **Range** | | **Mean (SD)** | |  |  |
|  | **Min Cutoff** | **Max Cutoff** | **TP** | **FP** | **TP** | **FP** | **Statistic** |  |
| **3D Entropy** | 7 | 1070 | [5.06, 6.88] | [4.43, 7.88] | 5.85 (0.41) | 6.74 (0.58) | F = 122, p < 0.001 |  |
| **2D Entropy** | 0 | 1581 | [4.18, 5.60] | [4.36, 7.49] | 5.01 (0.33) | 5.87 (0.66) | F = 86.4, p < 0.001 |  |
|  |  |  |  |  |  |  |  |  |
| 1. **SWI Frangi Filtered Blob Volume** | | | | | | | | |
|  | **False Positives Eliminated** | | **Range** | | **Mean (SD)** | |  |  |
| **Threshold** | **Min Cutoff** | **Max Cutoff** | **TP** | **FP** | **TP** | **FP** | **Statistic** |  |
| **0.10** | 142 | 638 | [511, 5023] | [1, 11186] | 1313 (695) | 3731 (2066) | F = 70.8, p < 0.001 |  |
| **0.15*** | 191 | 1164 | [415, 3087] | [1, 10350] | 1245 (475) | 3003 (1794) | F = 49.7, p < 0.001 |  |
| **0.20** | 260 | 966 | [339, 2668] | [1, 9149] | 1149 (354) | 2279 (1442) | F = 31.8, p < 0.001 |  |
| **0.25** | 325 | 945 | [278, 2149] | [1, 7751] | 984 (242) | 1747 (1166) | F = 22.3, p < 0.001 |  |
| **0.30** | 381 | 932 | [234, 1727] | [1, 6337] | 851 (198) | 1363 (951) | F = 15.1, p < 0.001 |  |
| **0.35** | 465 | 1030 | [195, 1265] | [1, 5288] | 738 (157) | 1080 (808) | F = 9.32, p < 0.01 |  |
| **0.40*** | 519 | 981 | [146, 1035] | [1, 3857] | 634 (138) | 859 (690) | F = 5.57, p < 0.05 |  |
| **0.45** | 0 | 953 | [1, 821] | [1, 2721] | 523 (160) | 682 (589) | F = 3.88, p < 0.05 |  |
| **0.50** | 283 | 941 | [9, 617] | [1, 2293] | 440 (123) | 535 (504) | F = 1.89, p = 0.17 |  |
| **0.55** | 0 | 787 | [1, 546] | [1, 2038] | 357 (118) | 409 (427) | F = 0.83, p = 0.36 |  |
| **0.60** | 0 | 622 | [1, 482] | [1, 1790] | 268 (124) | 301 (350) | F = 0.54, p = 0.46 |  |
| 1. **SWI Frangi Filtered Blob Compactness** | | | | | | | | |
|  | **False Positives Eliminated** | | **Range** | | **Mean (SD)** | |  |  |
| **Threshold** | **Min Cutoff** | **Max Cutoff** | **TP** | **FP** | **TP** | **FP** | **Statistic** |  |
| **0.10** | 115 | 480 | [309, 3743] | [1, 8057] | 557 (561) | 2413 (1427) | F = 87.2, p < 0.001 |  |
| **0.15*** | 176 | 936 | [314, 2293] | [1, 7288] | 540 (414) | 2060 (1287) | F = 72.0, p < 0.001 |  |
| **0.20** | 266 | 821 | [329, 1972] | [1, 6367] | 631 (322) | 1647 (1067) | F = 46.8, p < 0.001 |  |
| **0.25** | 329 | 658 | [276, 1762] | [1, 6022] | 724 (223) | 1323 (877) | F = 24.0, p < 0.001 |  |
| **0.30** | 388 | 597 | [234, 1525] | [1, 5241] | 714 (189) | 1072 (721) | F = 12.8, p < 0.001 |  |
| **0.35** | 467 | 896 | [195, 1140] | [1, 4664] | 668 (150) | 879 (624) | F = 5.93, p < 0.05 |  |
| **0.40*** | 520 | 874 | [146, 968] | [1, 3462] | 601 (133) | 725 (548) | F = 2.64, p = 0.10 |  |
| **0.45** | 0 | 887 | [1, 787] | [1, 2440] | 509 (156) | 596 (485) | F = 1.69, p = 0.19 |  |
| **0.50** | 283 | 896 | [9, 615] | [1, 2133] | 435 (121) | 482 (431) | F = 0.65, p = 0.42 |  |
| **0.55** | 0 | 754 | [1, 546] | [1, 1808] | 355 (118) | 379 (378) | F = 0.23, p = 0.63 |  |
| **0.60** | 0 | 602 | [1, 482] | [1, 1490] | 267 (123) | 285 (319) | F = 0.20, p = 0.65 |  |

**Supplementary Table S3. A.** Entropy cutoffs and distributions for GRE images across true positives (TP) and false positives (FP). **B.** Volume of central blobs in Frangi-filtered ROIs on GRE images across different thresholds. Note that the cutoffs chosen were based on the distributions that gave the maximum difference between true positives and false positives. **C.** Compactness of central blobs in Frangi-filtered ROIs on GRE images across different thresholds. (*marks the threshold values selected, 0.15 for min cutoff, 0.25 for max cutoff)

| 1. **GRE Entropy** | | | | | | | |
| --- | --- | --- | --- | --- | --- | --- | --- |
|  | **False Positives Eliminated** | | **Range** | | **Mean (SD)** | |  |
|  | **Min Cutoff** | **Max Cutoff** | **TP** | **FP** | **TP** | **FP** | **Statistic** |
| **3D Entropy** | 6 | 870 | [5.07, 6.88] | [4.67, 7.92] | 5.79 (0.38) | 6.63 (0.57) | F = 123, p < 0.001 |
| **2D Entropy** | 8 | 861 | [4.46, 5.89] | [4.28, 7.56] | 5.01 (0.31) | 5.67 (0.62) | F = 62.9, p < 0.001 |
| 1. **GRE Frangi Filtered Blob Volume** | | | | | | | |
|  | **False Positives Eliminated** | | **Range** | | **Mean (SD)** | |  |
| **Threshold** | **Min Cutoff** | **Max Cutoff** | **TP** | **FP** | **TP** | **FP** | **Statistic** |
| **0.10** | 216 | 238 | [231, 2938] | [1, 8819] | 720 (467) | 1506 (1072) | F = 30.0, p < 0.001 |
| **0.15*** | 245 | 154 | [60, 2333] | [1, 7822] | 493 (344) | 1010 (813) | F = 22.6, p < 0.001 |
| **0.20** | 0 | 352 | [1, 1284] | [1, 6714] | 318 (211) | 669 (596) | F = 19.4, p < 0.001 |
| **0.25*** | 0 | 1071 | [1, 444] | [1, 4911] | 192 (121) | 438 (442) | F = 17.3, p < 0.001 |
| **0.30** | 92 | 840 | [2, 368] | [1, 2757] | 129 (99) | 294 (337) | F = 13.3, p < 0.001 |
| **0.35** | 0 | 696 | [1, 287] | [1, 2272] | 84 (80) | 198 (257) | F = 10.9, p < 0.01 |
| **0.40** | 0 | 534 | [1, 237] | [1, 1417] | 59 (66) | 134 (189) | F = 8.81, p < 0.01 |
| **0.45** | 0 | 443 | [1, 189] | [1, 1202] | 41 (52) | 90 (142) | F = 6.74, p < 0.01 |
| **0.50** | 0 | 344 | [1, 153] | [1, 1047] | 32 (40) | 61 (106) | F = 4.10, p < 0.05 |
| **0.55** | 0 | 323 | [1, 101] | [1, 926] | 18 (25) | 40 (77) | F = 4.81, p < 0.05 |
| **0.60** | 0 | 253 | [1, 79] | [1, 788] | 12 (17) | 26 (55) | F = 3.16, p = 0.058 |
| 1. **GRE Frangi Filtered Blob Compactness** | | | | | | | |
|  | **False Positives Eliminated** | | **Range** | | **Mean (SD)** | |  |
| **Threshold** | **Min Cutoff** | **Max Cutoff** | **TP** | **FP** | **TP** | **FP** | **Statistic** |
| **0.10** | 216 | 217 | [231, 2524] | [1, 6170] | 577 (395) | 1241 (899) | F = 30.4, p < 0.001 |
| **0.15*** | 245 | 119 | [60, 2197] | [1, 5566] | 456 (309) | 884 (697) | F = 21.0, p < 0.001 |
| **0.20** | 0 | 273 | [1, 1205] | [1, 5307] | 307 (198) | 610 (523) | F = 18.8, p < 0.001 |
| **0.25*** | 0 | 1069 | [1, 432] | [1, 4169] | 190 (117) | 412 (399) | F = 17.2, p < 0.001 |
| **0.30** | 92 | 860 | [2, 356] | [1, 2444] | 129 (97) | 282 (314) | F = 13.3, p < 0.001 |
| **0.35** | 0 | 695 | [1, 287] | [1, 2096] | 84 (80) | 192 (243) | F = 11.0, p < 0.001 |
| **0.40** | 0 | 533 | [1, 237] | [1, 1156] | 59 (66) | 131 (181) | F = 8.92, p < 0.01 |
| **0.45** | 0 | 441 | [1, 189] | [1, 988] | 41 (52) | 89 (137) | F = 6.84, p < 0.01 |
| **0.50** | 0 | 344 | [1, 153] | [1, 901] | 32 (40) | 60 (103) | F = 4.15, p < 0.05 |
| **0.55** | 0 | 323 | [1, 101] | [1, 707] | 18 (25) | 40 (75) | F = 4.95, p < 0.05 |
| **0.60** | 0 | 253 | [1, 79] | [1, 645] | 12 (17) | 26 (54) | F = 3.71, p = 0.054 |
